# The Level of Unmet Need for Family Planning and Its Predictors among HIV-Positive Women in Ethiopia: A Systematic Review and Meta-Analysis

**DOI:** 10.1155/2021/3139272

**Published:** 2021-01-21

**Authors:** Maru Mekie, Dagne Addisu, Wubet Taklual, Abenezer Melkie

**Affiliations:** ^1^Department of Midwifery, College of Health Sciences, Debre Tabor University, Debre Tabor, Ethiopia; ^2^Department of Public Health, College of Health Sciences, Debre Tabor University, Debre Tabor, Ethiopia

## Abstract

**Background:**

Studies indicated that the need for family planning appears to be greater for human immuno-deficiency virus- (HIV-) positive women than the general population to reduce the risk of pediatrics HIV infection and related consequences of unintended pregnancy. We aimed to assess the level of unmet need for family planning and its predictors among HIV-positive women in Ethiopia.

**Methods:**

Online databases such as PubMed, SCOPUS, EMBASE, HINARI, Google Scholar, and digital libraries of universities were used to search for studies to be included in this systematic review and meta-analysis. Quality assessment of included studies was conducted using the Newcastle-Ottawa Quality Assessment Scale (NOS). Data were extracted using the format prepared on Excel workbook and analyzed by the Stata 11 software. Cochran (*Q* test) and *I*^2^ test statistics were used to assess the heterogeneity of studies. Similarly, the funnel plot and Egger's regression asymmetry test were used to assess publication bias.

**Result:**

This systematic review and meta-analysis was conducted using nine primary studies with a total of 6,154 participants. The pooled prevalence of unmet need for family planning among HIV-positive women was found to be 25.72% (95% CI: 21.63%, 29.81%). Participants age 15-24 years ((OR = 3.12; 95% CI: 1.59, 6.11) *I*^2^ = 27.5%; *p* = 0.252), being illiterate ((OR = 2.69; 95% CI: 1.69, 4.26) *I*^2^ = 0.0%; *p* = 0.899), failure to discuss FP with partner ((OR = 3.38; 95% CI: 2.20, 5.18) *I*^2^ = 0.0%; *p* = 0.861), and no access to family planning information ((OR = 4.70; 95% CI: 2.83, 7.81) *I*^2^ = 0.0%; *p* = 0.993) were found to be a significant predictors of unmet need for family planning among HIV-positive women.

**Conclusion:**

The level of unmet need for family planning among HIV-positive women was found to be high in Ethiopia. Being young age, illiteracy, failed to discuss family planning issues with a partner, and no access to family planning information were found to be the significant predictors of unmet need for family planning among HIV-positive women in Ethiopia. Improving information access and encouraging partners' involvement in family planning counseling and services could reduce the level of unmet need for family planning.

## 1. Background

Unmet need for family planning is defined as the proportion of women who do not want to have more children or want to delay the next child for at least two years but are not currently using contraception [1, 2]. According to the United Nations, more than 10% of women worldwide reported as they want to stop or delay pregnancy but are not using any method of contraception [3]. The magnitude of the unmet need for family planning services is found to be high in developing countries. About 222 million women in developing countries reported having an unmet need for family planning services as reported by the World Health Organization (WHO) [4]. According to studies, the level of unintended pregnancy was found to be higher among HIV-positive women compared to the general population [5–7]. A study conducted in South Africa indicated that half and one-third of pregnancies were unintended pregnancies among HIV-positive and HIV-negative women, respectively [6]. Another study conducted in the United States of America indicated that 60% of births among HIV-positive women were unplanned births which could be through the provision of family planning services [5]. A high rate of unintended pregnancy might increase the risk of unsafe abortion, mother to child transmission of HIV, maternal morbidity, and mortality.

Evidence indicated that the need for family planning appears to be greater for HIV-positive women compared to the general population to reduce pediatrics HIV-positive rates, orphans, and related consequences [8, 9]. Contraceptive use is also essential to reduce costs associated with HIV/AIDS care and treatment. However, the met need for family planning is found to be lower among HIV-infected women [7, 10, 11]. Family planning can improve the health of women by delaying first births, lengthening birth intervals, reducing high-risk pregnancies, and reducing unintended pregnancies that could lead to unsafe abortions [4].

Despite the demand for family planning is found to be high among HIV-positive women, the level of use is variable across areas and times. Contraceptive use initially increases among women in response to learning their HIV status and receiving counseling. However, the utilization of family planning declines with time in settings with low contraceptive use and high fertility [2, 11].

Improving the integration of family planning services with HIV prevention and treatment and other programs provides the best opportunity to increase family planning service uptakes among HIV-positive women which reduces the rate of unintended pregnancy and infected pediatrics population [4]. A study conducted among HIV-positive women in the Amhara region indicated that the level of HIV family panning integration was found to be very low. The study indicated that among HIV-positive women who reported using family planning services, only 1.1% got the service at ART clinic [12]. Different family planning service delivery mechanisms are implemented in Ethiopia including clinic-based, social marketing, and community-based distribution to increase family planning service utilization in the country [13].

Despite there is a substantial reduction in the level of unmet need for family planning from 37% in 2000 to 22% in 2016, the level of unmet need is still high in Ethiopia [14, 15]. According to the 2016 Ethiopian Demographic and Health Survey (EDHS), the level of unmet need for family planning services was reported to be 22% for the general population. There was a significant variation in the level of unmet need for family planning among reproductive-age women between regions, ranges from 11% to 29% in Addis Ababa City and Oromiya Region, respectively [14]. The analysis of the EDHS reports indicated that sociodemographic factors such as education level, partner education, religion, household wealth, number of living children in residence, and media exposure were found to be predictors of unmet need for family planning among reproductive-age women [15]. On the other hand, studies conducted in different countries indicated that desire to have children, husband support [16], women age [17, 18], low household income [18], parity, and previous use of family were found to affect the level of unmet need for family planning among HIV-positive women [17]. However, the level of unmet need for family planning services and its determinant factors among HIV-positive women is not known at the national level apart from individual studies conducted in different parts of the country with significant variation in the prevalence of unmet need for family planning [12, 19–26]. To the best of the authors' knowledge, this systematic review and meta-analysis is the first to be conducted about the level of unmet need for family planning services and its predictors among HIV-positive women.

## 2. Methods

### 2.1. Study Design

All studies conducted about the level of unmet need for family planning services among HIV positive women in Ethiopia were included. Hence, the available and included studies for this systematic review and meta-analysis were cross-sectional studies.

### 2.2. Search Strategy and Study Selection

Published peer-reviewed articles and unpublished studies were searched using electronic databases and digital libraries of universities. Following the quality assessment of the studies, a systematic review and meta-analysis was conducted to assess the level of unmet need for family planning and its predictors among HIV-positive women in Ethiopia.

**Figure 1:**
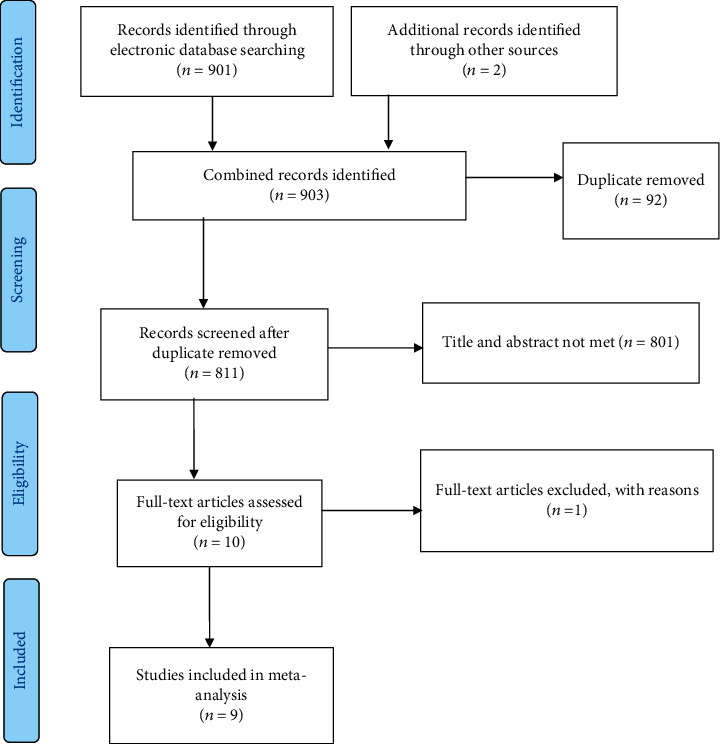
PRISMA flow chart indicating study selection for systematic review and meta-analysis of unmet need for family planning among HIV-positive women in Ethiopia.

Candidate studies reported in the English language were identified through an online search of different databases such as PubMed, SCOPUS, EMBASE, HINARI, and Google Scholar, and digital libraries of universities were used to search studies to be included in this systematic review and meta-analysis. We have used PICO (P = population, I = intervention, C = comparison, and O = outcome) to formulate search terms in identifying the candidate studies [27]. P is the HIV-positive reproductive-age women, I is the family planning, O is the level of unmet need, and C is the comparison of the level of met need for family planning. The search terms used were unmet need, unmet demand, family planning services, HIV positive, seropositive, women, associated factors, determinants, AND Ethiopia (Table 1). Article search was conducted from July 25 to August 1/2020. Selection and exclusion of studies for the systematic review and meta-analysis was presented using the Preferred Reporting Items for Systematic Reviews and Meta-Analysis (PRISMA) guidelines [28] (Figure 1).

### 2.3. Inclusion and Exclusion Criteria

#### 2.3.1. Inclusion Criteria

Studies reporting prevalence and factors associated with unmet need for family planning among HIV-positive women published from January 2005 to August 1, 2020 or unpublished studies conducted from January 2005 to August 1, 2020 reporting an unmet need for family planning among HIV-positive women in Ethiopia were included in this systematic review and meta-analysis. All studies conducted in Ethiopia about the level of unmet need for family planning services among HIV-positive women reported in English language during the stated period were included in this systematic review and meta-analysis.

#### 2.3.2. Exclusion Criteria

With reference to the exclusion criteria, studies that did not report the outcome of interest and those not fully accessible after two email contacts for corresponding authors were excluded.

### 2.4. Data Abstraction

MM has prepared the data abstraction format using Microsoft Excel workbook to extract data on the level of unmet need for family planning and predictor variables among HIV-positive women. Author name, publication year, study region, study site, study design, study period, sample size, prevalence, and standard error of the prevalence were included in the Excel worksheet for data abstraction. Moreover, the abstraction form also includes factors affecting the unmet need for family planning among HIV-positive women with their respective odds ratio, logor, and standard error of the logor.

### 2.5. Measurement of the Outcome Variable

Unmet need for family planning is defined as the proportion of women who do not want to have more children or want to delay the next child for at least two years but are not currently using contraception [1, 2].

### 2.6. Data Quality Assurance and Data Analysis

Two authors, MM and DA, independently reviewed the titles and abstracts of studies. After the studies were exported to endnote 7, duplicates were managed. Disagreements on inclusion and exclusion of studies were resolved through discussion including the third (WT) and the fourth (AM) authors. The Newcastle-Ottawa Quality Assessment Scale (NOS) was used to critically appraise the studies for inclusion in this systematic review and meta-analysis. The criteria used to critically appraise the studies for inclusion in this systematic review and meta-analysis were as follows: (1) representativeness of the sample, (2) sample size, (3) nonrespondents, (4) ascertainment of the risk factor, (5) the subjects in different outcome groups are comparable and control of confounding factors, (6) assessment of the outcome, and (7) the statistical test used. Hence, studies with quality scores of ≥7 were considered as low risk for bias in the NOS (Table 2).

The extracted data were exported to the Stata version 11 software from the Excel worksheet for further analysis. The funnel plot and Egger's regression test were used to assess publication bias through observation and *p* value with its 95% CI, respectively. The pooled prevalence of unmet need for family planning services and its determinant factors among HIV-positive women were presented using a forest plot with a 95% confidence level. The random variation between primary studies was assessed using Cochran (*Q* test) and *I*^2^ test [29]. Heterogeneity was interpreted as an *I*^2^ value = 0% as no heterogeneity, 25% = low heterogeneity, 50% = moderate heterogeneity, and 75% = high heterogeneity in this systematic review and meta-analysis [30]. Radom model was used to estimate the pooled prevalence of unmet need for family planning services and its predictors by assuming the existence of variability between and within studies.

## 3. Results

A total of 903 studies were retrieved through online databases including digital libraries of universities. Ninety-two studies were excluded due to duplicates, and 801 studies were excluded after review of titles and abstracts giving 10 candidate studies for inclusion. One study was excluded due to reason (not being fully accessible) giving 9 full articles for final inclusion in this systematic review and meta-analysis (Figure 1).

### 3.1. Characteristics of Included Studies

Nine studies with a total of 6,154 participants published from January 1, 2005, to August 1, 2020, were included to determine the level of unmet need for family planning among HIV-positive women in Ethiopia. With regard to study regions, the included studies were conducted in Amhara region, Addis Ababa City administration, Oromiya region, Southern Nations Nationalities and People (SNNP) Region, and Dire Dawa City administration.

The highest prevalence of unmet need was reported in a study conducted at health institutions in Dire Dawa by Kassa et al. (36.07%) [24] followed by a study conducted in Amhara region referral hospitals by Zewdie et al. (35%) [12], while the lowest was reported in a study in Oromiya region by Feyissa and Melka (15.38%) [22] (Table 2).

## 4. Meta-Analysis

### 4.1. The Level of Unmet Need for Family Planning among HI-Positive Women

**Figure 2:**
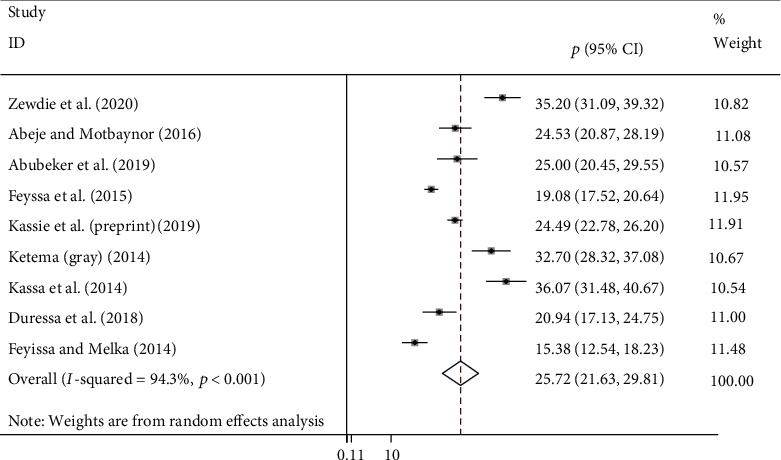
Forest plot revealing the pooled level of unmet need for family planning among HIV-positive women in Ethiopia.

Nine studies were used to estimate the pooled prevalence of unmet need for family planning among HIV-positive women in Ethiopia (Figure 2). Hence, the pooled level of unmet need for family planning among HIV-positive women was found to be 25.72% (95% CI: 21.63%, 29.81%).

### 4.2. Publication Bias

**Figure 3:**
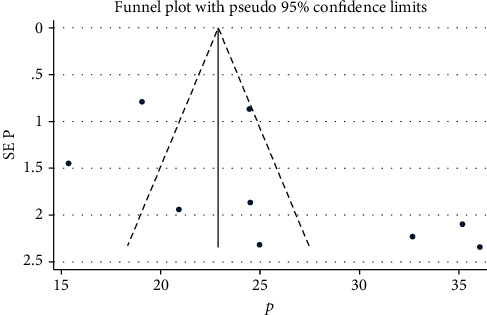
Funnel plot for assessing publication bias of the level of unmet need for family planning among HIV-women in Ethiopia.

Publication bias was assessed using funnel plot through visual observation and Egger's regression asymmetry test (Figure 3). The Egger's asymmetry test indicated that there was no evidence of publication bias with *p* value of 0.12 (95% CI: -1.52, 12.39).

### 4.3. Heterogeneity

**Figure 4:**
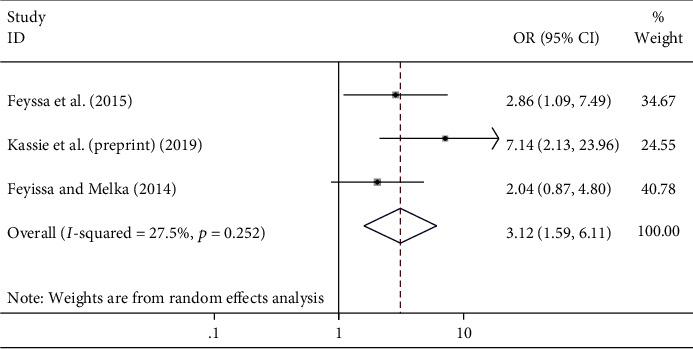
The association between age and unmet need for family planning among HIV-positive women in Ethiopia.

**Figure 5:**
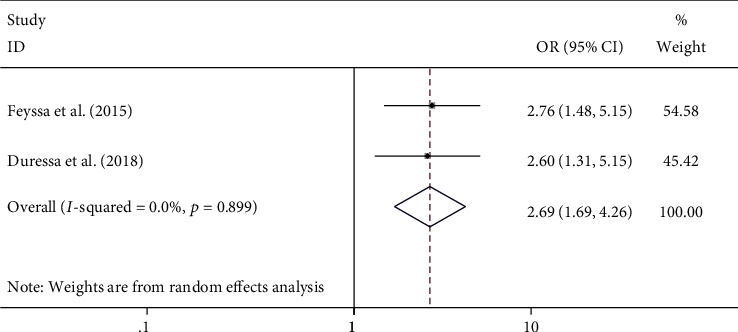
The association between illiteracy and unmet need for family planning among HIV-positive women in Ethiopia.

To assess heterogeneity between primary studies, subgroup analysis was conducted based on study regions and sample size (Figures 4 and 5).

### 4.4. Subgroup Analysis of the Prevalence of Unmet Need for Family Planning among HIV-Positive Women in Ethiopia

Subgroup analysis was performed based on the study regions and sample size to single out the cause of heterogeneity (Table 3). The subgroup analysis based on region indicated that the level of the unmet need for family planning services among HIV-positive women was found to be higher among women in Addis Ababa (28.87%) (95% CI: 21.33%, 36.42%) and Amhara region (27.88%) (95% CI: 21.85%, 33.925), whereas it was found to be lower in SNNPR (19.08%) (95% CI: 17.52%, 20.64%) and Oromiya region (18.01%) (95% CI: 12.58%, 23.45%). Significant heterogeneity was observed between studies following the subgroup analysis based on regions.

Similarly, the subgroup analysis of the level of unmet need for family planning among HIV-positive women based on sample size indicated that there was no significant difference in the level of unmet need for family planning among studies with a sample size ≥ 500 (24.80% (95% CI: 18.19%, 31.41%), *I*^2^ = 94.5% and 95.8%) and <500 (26.55% (95% CI: 20.17%, 32.93%), *I*^2^ = 95.8%) with significant heterogeneity between studies.

### 4.5. Factors Associated with Unmet Need for Family Planning among HIV-Positive Women in Ethiopia

In this meta-analysis, being younger age, illiteracy, no access to family planning information, and no discussion about family planning with partner were found to be the significant predictors of unmet need for family planning services among HIV-positive women. However, there was no statistically significant association between the desire of more children and an unmet need for family planning (Table 4).

#### 4.5.1. The Association between Age and Unmet Need for Family Planning

Being younger age [22, 23, 25] was found to be significantly associated with unmet need for family planning among HIV-positive women. The likely hood of experiencing an unmet need for family planning was found to be 3 times higher among women ages 15-24 years compared to those with ages ≥ 35 years ((OR = 3.12; 95% CI 1.59, 6.11) *I*^2^ = 27.5%; *p* = 0.252) (Figure 4).

#### 4.5.2. The Association between Education Level and Unmet Need for Family Planning among HIV-Positive Women

The education level of the study participants was found to be a significant predictor of having an unmet need for family planning service [21, 23]. The odds of having an unmet need for family planning were found to be 2.69 times higher among uneducated HIV-positive women compared with their counterparts ((OR = 2.69; 95% CI: 1.69, 4.26) *I*^2^ = 0.0%; *p* = 0.899) (Figure 5).

#### 4.5.3. The Association between Desire to Have More Children and Unmet Need for Family Planning

**Figure 6:**
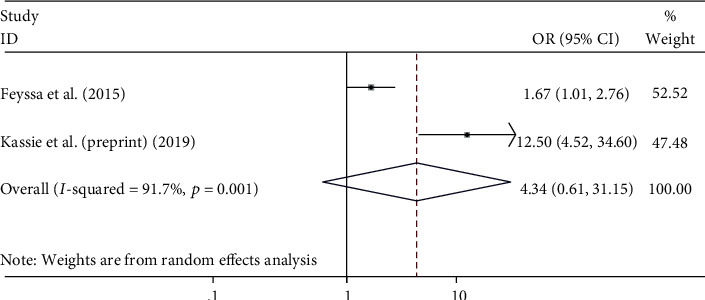
The association between desire of more children and unmet need for family planning among HIV-positive women in Ethiopia.

Statistical significant association was not found between a desire to have more children planning [23, 25] and the unmet need for family planning among HIV-positive women ((OR = 4.34, 95%, CI 0.61, 31.15), I^2=^ 91.7%, p = 0.001) (Figure 6).

#### 4.5.4. Discussion with Partner about Family Planning and Its Association with Unmet Need

**Figure 7:**
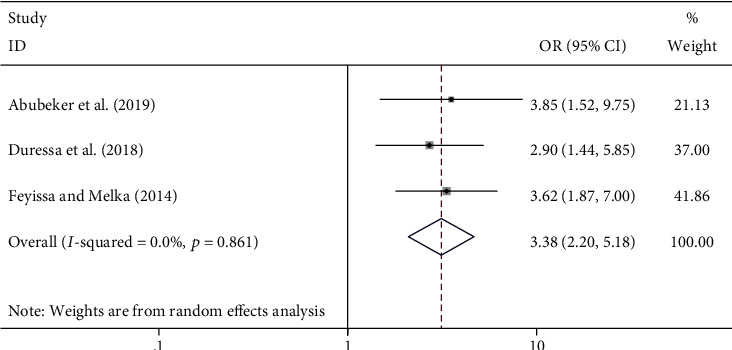
The association between failure to discussion about family planning with partner and unmet need for family planning among HIV-positive women in Ethiopia.

Failed to discuss about family planning issues with sexual partner [20–22] was found to be associated with the unmet need for family planning among HIV-positive women. The odds of reporting an unmet need for family planning services were found to be more than three times higher among women who failed to discuss family planning issues with partner compared with counterparts ((OR = 3.38; 95% CI: 2.20, 5.18) *I*^2^ = 0.0%; *p* = 0.861) (Figure 7).

#### 4.5.5. The Association between Having Access to Family Planning Information and Unmet Need for Family Planning

**Figure 8:**
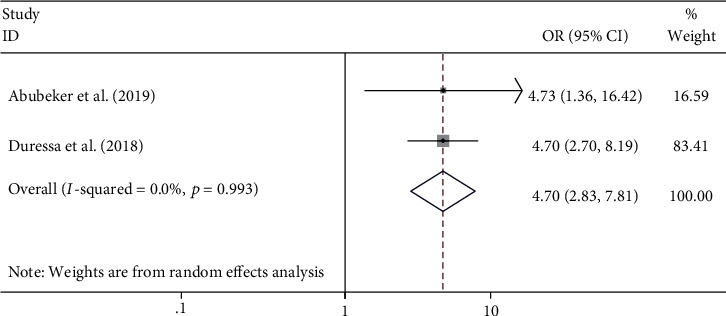
The association between no access to family planning information and unmet need for family planning among HIV-positive women in Ethiopia.

No access to family planning service was found to be associated with the likelihood of having unmet need for family planning among HIV-positive women [20, 21]. The odds of experiencing an unmet need for family planning were found to be 4.7 times higher among HIV-positive women who had no access to family planning information compared to counterparts ((OR = 4.70; 95% CI: 2.83, 7.81) *I*^2^ = 0.0%; *p* = 0.993) (Figure 8).

## 5. Discussion

This meta-analysis is aimed at assessing the level of unmet need for family planning among HIV-positive women and its predictors in Ethiopia. In this study, the level of unmet need for family planning among HIV-positive women in Ethiopia was found to be 25.72% ((95% CI: 21.63%, 29.81%) *I*^2^ = 94.3%; *p* < 0.001). A similar finding was reported in studies conducted in Kumasi, Ghana [31], and Bandung City, Indonesia [16], which reported unmet needs of 27.8% and 27.7%, respectively [16, 31]. The level of unmet need for family planning among HIV-positive women in our study was found to be higher than the level of unmet need among the general population which reported 22% as of Ethiopian Demographic and Health Survey (EDHS) [14]. The finding of our study implies that the demand for family planning among HIV-positive women is not addressed well despite its relevance in reducing pediatrics HIV rates in the country. On the other hand, the level of unmet need for family planning among HIV-positive women in our study was found to be lower than studies conducted in Nairobi, Kenya [32], and Uganda [18] which reported 33.6% and 38% of unmet need for family planning, respectively. The variation in the rate of unmet need might be attributed to the difference in health policy and health coverage of the countries.

The subgroup analysis of the level of unmet need family planning among HIV-positive women in our study indicated that the highest and the lowest level of unmet for family planning among HIV-positive women were found in Addis Ababa City administration and Oromiya region, respectively. The finding of our study is not supported by the EDHS report [14]. The reason for the variation might be related to the difference in the study population in which the EDHS report was conducted from the general reproductive-age population in contrast to our study which was conducted among HIV-positive women.

The age of the study participants was found to be a determinant factor for the unmet need for family planning service among HIV-positive women. Young women age 15-24 years were found to be 3.12 times more likely to have an unmet need for family planning compared to those ≥35 years (OR = 3.12; 95% CI: 1.59, 6.11). A similar finding was reported in a study conducted in Nigeria which reported low unmet need among women ages 35-45 years compared to women ages 18-24 years [17]. The higher level of unmet need for family planning among young women might be related to poor access and low awareness of where and how to get family planning services.

Similarly, the education level of the study participants was found to be significantly associated with the unmet need for family planning among HIV-positive women in this meta-analysis. The likely hood of experiencing unmet need was found to be 2.69 times higher among illiterate women compared with counterparts (OR = 2.69; 95% CI: 1.69, 4.26). A similar finding was reported in a study conducted in Nairobi, Kenya [32]. Improving access to family planning services to the community to the nearest possible area using different mechanisms such as social marketing and community-based distribution is significant to reduce the level of unmet need for family planning services.

Discussion with partner regarding family planning issues was found to be the determinant of the level of unmet need for family planning. The odds of having an unmet need for family planning were found to be 3.38 times higher among women who failed to discuss family planning issues with sexual partners compared to their counterparts (OR = 3.38; 95% CI: 2.20, 5.18). The finding of our study is supported by studies conducted in India [33] and Indonesia [34] which reported discussion on family planning issues with a sexual partner and husband support as important factors to reduce unmet need for family planning services. The finding of our study indicated that male involvement in the discussion of reproductive health issues is imperative to reduce the level of unmet need. Hence, joint decisions between the women and sexual partners shall be strengthened to reduce the level of unmet need for family planning.

The odds of having an unmet need for family planning were found to be 4.7 times higher among women who did not have access to family planning information compared to those who had access to information (OR = 4.70; 95% CI: 2.83, 7.81). The finding of this systematic review and meta-analysis indicated that improving access to family planning information and services could significantly reduce the level of unmet need for family planning among HIV-positive women. A similar finding was reported in a study conducted in Kenya [32].

Statistically, a significant association was not found between the desire of more children and unmet need in this meta-analysis (OR = 4.34; 95% CI: 0.61, 31.15) despite significant association was reported in the individual studies. The finding of this study is not supported by studies conducted in Indonesia [16] and India [33] which found a high unmet need and low utilization of family planning among HIV-positive women who had a desire to have more children. The discrepancy between our study and the aforementioned studies might be due to the limited number of included studies to assess the association between the desire for more children and the unmet need for family planning among HIV-positive women.

### 5.1. Limitations of the Study

Despite this systematic review and meta-analysis is the first to be conducted among HIV-positive women, it has its limitations to be considered in the interpretation of the finding. Due to the nonavailability of studies, the systematic review and meta-analysis was conducted by using limited numbers of studies. Similarly, we failed to find studies conducted on the level of unmet need for family planning among HIV-positive women in all the corners of the country which might affect generalization.

## 6. Conclusion

The level of unmet need for family planning among HIV-positive women was found to be high in Ethiopia compared with the general population. Being young age, illiteracy, failure to discuss family planning issues with a partner, and no access to family planning information were found to be significant predictors of unmet need for family planning among HIV-positive women in Ethiopia. Improving information access to family planning and encouraging partner's involvement in family planning counseling and services would reduce the level of unmet need for family planning among HIV-positive women.

## Figures and Tables

**Table 1:** Search strategies used for PubMed and related databases to assess candidate studies for the systematic review and meta-analysis.

Data bases	Search terms	Number of studies
PubMed/Medline	unmet[All Fields] AND need[All Fields] OR “Unmet demand”[all fields] AND (“family planning services”[MeSH terms] OR (“family”[All Fields] AND “planning”[All Fields] AND “services”[All Fields]) OR “family planning services”[All Fields] OR (“family”[All Fields] AND “planning”[All Fields]) OR “family planning”[All Fields]) AND (“hiv seropositivity”[MeSH Terms] OR (“hiv”[All Fields] AND “seropositivity”[All Fields]) OR “hiv seropositivity”[All Fields] OR (“hiv”[All Fields] AND “positive”[All Fields]) OR “hiv positive”[All Fields]) AND (“women”[MeSH Terms] OR “women”[All Fields]) AND associated[All Fields] AND factors[All Fields] OR “predictors”[all] OR “determinant factors “[All Fields] AND fields[All Fields] AND (“ethiopia”[MeSH Terms] OR “ethiopia”[All Fields]).	803
Other databases		98
Gray literature		2
Total search		903
Numbers candidate for inclusion		10
Excluded with reasons		1
Studies included in the analysis		9

**Table 2:** Characteristics of studies included in the systematic review and meta-analysis of unmet need for family planning among HIV-positive women in Ethiopia.

Author	Publication year	Region	Study area	Study design	Sample size	Population of outcome	Prevalence	Response rate	Risk of bias
Zewdie et al. [12]	2020	Amhara	Amhara referral H.	Cross-sectional	517	182	35.20	100%	Low risk
Abeje and Motbaynor [19]	2016	Amhara	Health facilities	Cross-sectional	530	130	24.53	100%	Low risk
Abubeker et al. [20]	2019	Addis Ababa	Paul's hospital	Cross-sectional	348	87	25.00	95.90%	Low risk
Feyssa et al. [23]	2015	SNNPR	Hawassa ref hospital	Cross-sectional	2,442	466	19.08	99.60%	Low risk
Kassie et al. (preprint) [25]	2019	Amhara	Gondar city	Cross-sectional	441	108	24.49	100%	Low risk
Ketema (gray) [26]	2014	Addis Ababa	Zewuditu hospital	Cross-sectional	419	137	32.70	98.45	Low risk
Kassa et al. [24]	2014	Dire Dawa	Health facilities	Cross-sectional	438	158	36.07	100%	Low risk
Duressa et al. [21]	2018	Oromiya	Sibu Sire district	Cross-sectional	616	129	20.94	100%	Low risk
Feyissa and Melka [22]	2014	Oromiya	Nekemte hospi and HC	Cross-sectional	403	62	15.38	99.5	Low risk

**Table 3:** Subgroup analysis of the pooled level of unmet need of family planning among HIV-positive women in Ethiopia.

Category	Numbers of studies	Prevalence with (95% CI, *I*^2^, and *Q* value)	Model	Heterogeneity
Study regions				
Amhara region	3	(27.88 (21.85, 33.92), *I*^2^ = 91.2%, *p* < 0.001)	Random	Significant heterogeneity
Addis Ababa	2	(28.87 (21.33, 36.42), *I*^2^ = 82.5%, *p* = 0.017)	Random	Significant heterogeneity
SNNPR	1	(19.08 (17.52, 20.64), *I*^2^ = --, *p* = --)	--	--
Dire Dawa	1	(36.07 (31.48, 40.67), *I*^2^ = --, *p* = --)	--	--
Oromiya region	2	(18.01 (12.58, 23.45), *I*^2^ = 80.9%, *p* = 0.022)	Random	Significant heterogeneity
Sample size				
≥500	4	(24.80 (18.19, 31.41), *I*^2^ = 94.5%, *p* < 0.001)	Random	Significant heterogeneity
<500	5	(25.72 (21.63, 29.81), *I*^2^ = 94.3%, *p* < 0.001)	Random	Significant heterogeneity

**Table 4:** Summary of variables associated with unmet need for family planning among HIV-positive women in Ethiopia.

Variables	Numbers of studies	OR with 95% confidence interval	*I* ^2^	*p* value
Being 15-24 years of age	3	(OR = 3.12; 95% CI: 1.59, 6.11)	*I* ^2^ = 27.5%,	*p* = 0.252
Women with no education	2	(OR = 2.69; 95% CI: 1.69, 4.26)	*I* ^2^ = 0.0%	*p* = 0.899
Desire to have more children	2	(OR = 4.34; 95% CI: 0.61, 31.15)	*I* ^2^ = 91.7%	*p* = 0.001
No discussion with partner	3	(OR = 3.38; 95% CI: 2.20, 5.18)	*I* ^2^ = 0.0%	*p* = 0.861
No access to family planning	2	(OR = 4.70; 95% CI: 2.83, 7.81)	*I* ^2^ = 0.0%	*p* = 0.993

## Data Availability

All relevant data are presented within the manuscript. The dataset used to reach conclusion can be assessable from the corresponding author on request.

## Abbreviations

AIDS: Acquired immuno-deficiency syndrome
EDHS: Ethiopia Demographic and Health Survey
HIV: Human immuno-deficiency virus
NOS: Newcastle-Ottawa Quality Assessment Scale
OR: Odds ratio
WHO: World Health Organization.
